# Glycyrrhizinic Acid and *Isatidis folium* extracts combined with *Bacillus subtilis* protect against dextran sulfate sodi-um-induced colitis in Hu sheep

**DOI:** 10.1371/journal.pone.0362464

**Published:** 2026-11-10

**Authors:** Dianzhong Zheng, Xiao Fu, Manyu Li, Yufeng Liu, Di Liu

**Affiliations:** 1 Postdoctoral Workstation of Heilongjiang Academy of Agricultural Science, Harbin, Heilongjiang, China; 2 Institute of Animal Husbandry, Heilongjiang Academy of Agricultural Sciences, Harbin, Heilongjiang, China; 3 College of Electrical and Information, Northeast Agricultural University, Harbin, Heilongjiang, China; Suez University Faculty of Fish Resources, EGYPT

## Abstract

Weaning stress caused by maternal separation and dietary changes often leads to intestinal disorders such as diarrhea and enteritis in young animals, and the development of antibiotic-free, safe preparations to manage these conditions is therefore a promising strategy. The optimal proportion of each component in the formulation was determined in our preliminary study using orthogonal experimental design and range analysis. This study first evaluated the effects of glycyrrhizic acid and *Isatidis folium* extract on the growth of *Bacillus subtilis* (*B. subtilis*), and then examined the effects of the compound preparation (CP) composed of glycyrrhizic acid, *Isatidis folium* extract, and *B. subtilis* on in vitro antibacterial activity, growth performance, immune modulation, and the amelioration of dextran sulfate sodium (DSS)-induced colitis in Hu sheep. Twenty-seven 45-day-old healthy male Hu lambs (body weight = 15.83 ± 0.41 kg) were randomly assigned to 3 dietary groups, each dietary group consisted of 9 animals, were housed in 3 pens with 3 sheeps in each. The lambs were fed with the basal diets (control group), supplemented with 25 g (compound preparation group 1, CP1), 30 g (compound preparation group 2, CP2) CP per kg diet, respectively. Experimental design of protective effects of CP on lamb colitis was eighteen 45-days-old healthy male Hu lambs (body weight = 16.24 ± 0.86 kg) were randomly assigned to three groups (n = 6 per group), the lambs were fed with the basal diets (control group); lambs were fed with the basal diets for the first 7 days and were then given a single daily oral gavage of DSS at 4 g/kg body weight (DSS group); lambs were fed with the basal diet supplemented with 25 g CP per kg diet for the first 7 days and were then given a single daily oral gavage of DSS at 4 g/kg body weight (CP-DSS). The results showed that glycyrrhizic acid and *Isatidis folium* extract did not affect the growth of *B. subtilis*, while the CP significantly inhibited the growth of *Escherichia coli* (*E. coli*) and *Staphylococcus aureus* (*S. aureus*). CP1 significantly increased daily weight gain; CP1 and CP2 significantly increased serum immunoglobulin G (IgG) and colonic mucosal secretory immunoglobulin A (SIgA) levels, as well as the relative mRNA expression of interferon γ (IFN-γ), interleukin-1β (IL-1β), interleukin-4 (IL-4), interleukin-6 (IL-6) and interleukin-10 (IL-10). Supplementation with 25 g CP per kg diet significantly downregulated the relative mRNA expression of proinflammatory cytokines (IFN-γ, IL-1β, IL-6) and upregulated the relative mRNA expression of anti-inflammatory cytokines (IL-4, IL-10) and intestinal barrier factors (ZO-1, occludin, Muc2, α-catenin), alleviating colonic damage. These findings support its potential as a safe and effective feed additives for promoting intestinal health in sheep.

## Introduction

Enteritis is a major health challenge in lambs [1], which commonly causes diarrhea, weight loss, and even hematochezia, leading to significant economic losses in the sheep industry each year [2]. Therefore, effective prevention and treatment of enteritis are essential. For decades, antibiotics have been widely administered to control intestinal disease. However, the extensive and often inappropriate use of antibiotics has contributed to the emergence of antimicrobial-resistant bacteria, posing risks to animal health, food safety, and public health [3]. In response, the European Union banned pro-growth antibiotics in 2006, while the European Union and the United States approved thousands of plant-derived extracts as feed additives [4,5]. China has also prohibited all growth-promoting antibiotic feed additives, except those derived from traditional Chinese medicine (TCM), since 2020 [6]. This shift highlights the urgent need for green, safe, and environmentally friendly feed additives instead of antibiotics.

*Isatidis folium*, derived from *Isatis indigotica*, has been used in TCM as an herbal remedy to treat various diseases for centuries [7]. *Isatidis folium* extracts, a common form used in clinical practice, exert detoxifying, antipyretic, and anti-inflammatory effects [8]. At present, researchers have confirmed the anti-inflammatory activity of *Isatidis folium* extract in specific experimental models. *Isatidis folium* extract can inhibit proinflammatory cytokines and chemokines by targeting the NF-κB signaling pathway, thereby alleviating skin inflammation in mice [9]. Moreover, *Isatidis folium* extracts can significantly enhance the activity of glutathione-related enzymes and reduce the levels of the toxic metabolite N-acetylbenzoquinone imide, thereby alleviating acetaminophen-induced liver injury in mice [10]. Our previous study found that *Isatidis folium* extract could modulate immunity in Hu sheep, exert no significant effects on routine blood parameters, and exhibit no detrimental impacts on Hu sheep [11].

Glycyrrhizic acid, an active component of Glycyrrhiza spp [12]., also exhibits diverse pharmacological activities, including anti-inflammatory [13], anti-tumor [14], antiviral [15], and immune-modulatory effects [16]. Glycyrrhizic acid mainly suppresses the nuclear factor kappa-B (NF-κB) signaling pathway, thereby further inhibiting the transcription of key genes, such as inflammatory cytokines, thereby achieving anti-inflammatory and anti-tumor effects [16]. Moreover, glycyrhizic acid has been found to significantly reduce the levels of tumor necrosis factor-α (TNF-α), IL-1β, and IFN-γ in the serum of broilers treated with lipopolysaccharide (LPS) [17]. Besides, glycyrrhizic acid can reduce cochlear permeability in mice and alleviate cisplatin-induced ototoxicity by slowing the inflammatory response [18]. Researchers found that feeding complex glycyrrhizin composed of phosphorylated glycyrrhizin and glycyrrhizic acid could effectively increase the body weight of broilers and the contents of IFN-γ, interleukin-2 (IL-2), interleukin-17 (IL-17), and intestinal secretory immunoglobulin A (SIgA) in broilers, thus exerting immune regulatory functions [19]. Our preliminary work found that glycyrrhizic acid promotes growth, improves antioxidant capacity and regulates immune function in Hu sheep [20].

*B. subtilis* can improve feed conversion efficiency, increase the nutritional value of the feed, enhance immunity, resist disease, and improve intestinal health [21]. Some researchers found that *B. subtilis*, when combined with *Bacillus licheniformis*, increased lamb weight, strengthened the immune system, and maintained normal metabolic processes [22]. A large number of microorganisms coexist in the gastrointestinal tract of ruminants, and the homeostasis of the gastrointestinal flora is crucial to the organism's health. *B. subtilis* can regulate animal intestinal flora and maintain intestinal microecological stability [23]. Recently, the combined use of TCM and probiotics has gained attention due to their complementary advantages, natural origin, low toxicity, minimal residues, and environmental safety. However, TCM or probiotics are still mostly practiced independently. When used independently, TCM may exhibit a slow onset, and some of its effective ingredients are poorly absorbed, limiting its widespread application. Probiotics, when used alone, may face stability challenges and a limited functional spectrum for disease control. A combined compound may overcome these limitations. Briefly, glycyrrhizic acid exerts immunomodulatory effects; *Isatidis folium* extract possesses anti‑inflammatory activity, and Bacillus subtilis improves intestinal barrier function and promotes intestinal health. When these three components are combined, they may act via complementary pathways. This complementary mode of action provides the rationale for testing the combined effect of the compound preparation

TCM and probiotics have been widely used for preventing and treating intestinal diseases due to their natural properties, low toxicity, low residue and no pollution. Nevertheless, most research adopts separate administration of TCMs or probiotics. TCM extracts alone exhibit limited efficacy and are difficult for practical application, while probiotics used individually suffer from poor stability and limited applicable scenarios. Therefore, we innovatively explored the combined effects of TCM extracts and probiotics on growth performance, immunity and colitis therapy in lambs to enhance their combined effect. In this study, a compound preparation consisting of glycyrrhizic acid, *Isatidis folium* extract and *B. subtilis* was developed to replace antibiotics for the prevention and treatment of diarrhea and enteritis in lambs. The effects of *Isatidis folium* extract and glycyrrhizic acid on the growth of *B. subtilis* were evaluated. The antibacterial activity against pathogenic bacteria was analyzed in vitro, and the efficacy of the preparation was then evaluated in vivo by assessing its effects on immune function, intestinal barrier function and alleviation of colitis injury in Hu sheep. This provides a reference for using this compound preparation as a safe and effective alternative to antibiotics to improve intestinal health and disease resistance in sheep.

## Materials and methods

All experimental procedures involving animals were approved (animal protocol number: IHA20250411) by the Animal Welfare and Ethics Committee of Institute of Animal Husbandry, Heilongjiang Academy of Agricultural Sciences, Harbin, Heilongjiang, China. Research staff involved in the study received systematic training, including training on laboratory animal regulations and operational standards. The schematic of the experimental design is shown in Fig 1.

**Fig 1:**
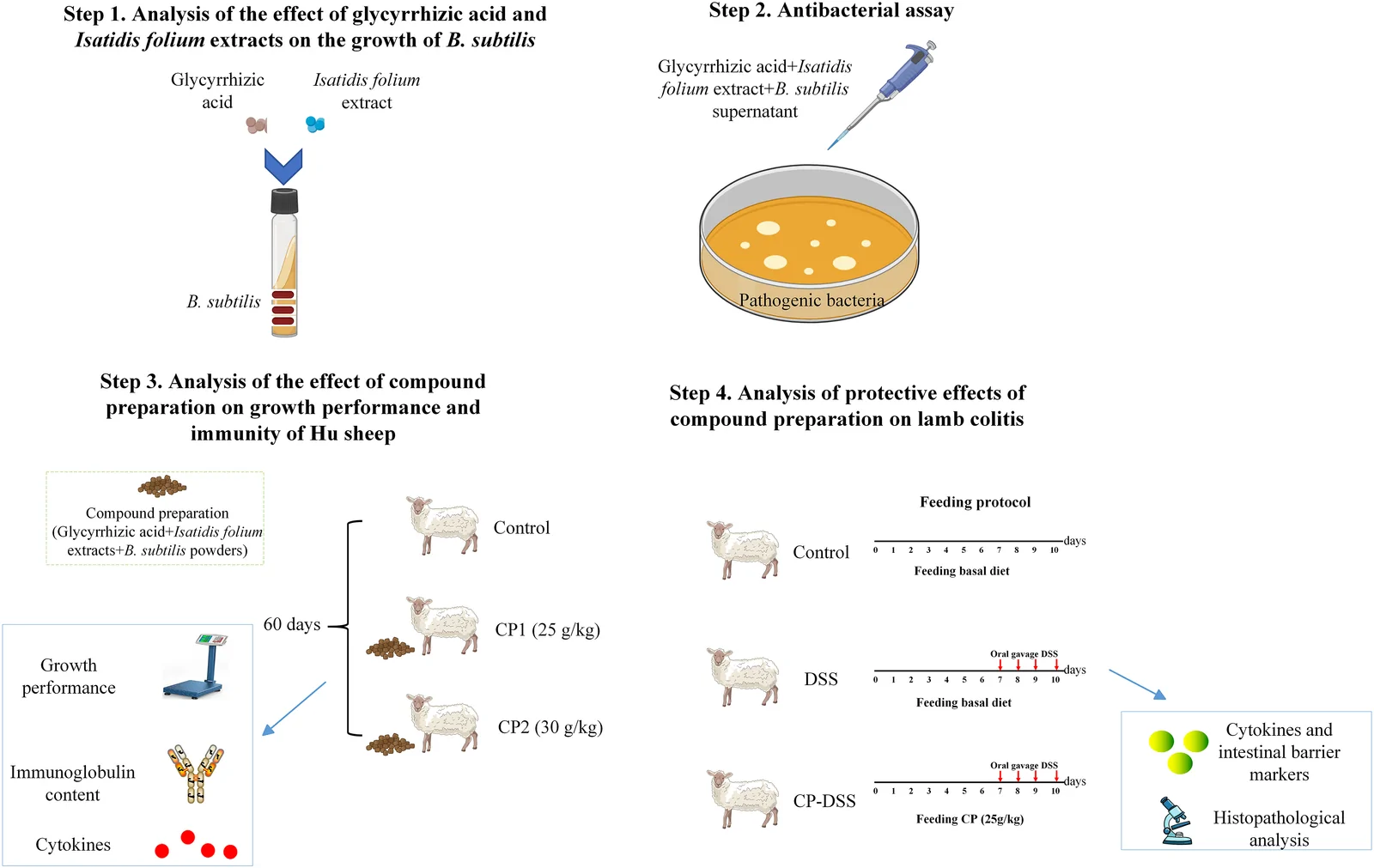
The graphical representation of the experimental design.

### Analysis of the effect of glycyrrhizic acid and *Isatidis folium* extracts on the growth of *B. subtilis*

Glycyrrhizic acid and *Isatidis folium* extract (major constituents comprising 10% flavonoids and 1% indirubin. Its extraction process consists of raw material crushing, extraction, filtration, concentration, purification and drying) were purchased from Mingyang Zhilian Biotechnology Co., Ltd. *Bacillus subtilis WB800N (B. subtilis) was purchased from Takara Biomedical Technology (Dalian) Co., Ltd., Dalian, China*. Activated *B. subtilis* cultures were adjusted to an OD_600_ of 1.0. A 1:100 inoculum was transferred into LB broth containing 5%, 10%, or 15% glycocyrrhizic acid and *Isatidis folium* extract. Cultures were shaken at 37 °C for 7 h. LB broth cultures without additives served as the control group. Each condition included three biological replicates. Bacterial growth was evaluated by measuring OD_600_.

### Antibacterial assay

Pathogenic *E. coli*, *Salmonella typhimurium* (*S. typhimurium*), and *S. aureus* were obtained from laboratory stock cultures. Glycyrrhizic acid and *Isatidis folium* extract were fully dissolved in deionized water to a final concentration of 100 mg/mL to prepare the complex, and the solution was sterilized at high temperature. *B. subtilis* was cultured in LB broth (Hopebio, China) at 37 °C for 12 h, and the cell-free supernatant was prepared by centrifugation at 10,000 × g for 10 min followed by filtration (0.22 µm pore size). The pathogenic *E. coli*, *S. aureus*, and *S. typhimurium* were cultured to an OD_600_ of 0.5, and 200 µL of each bacterial suspension was spread evenly on LB agar plates. Three wells (diameter 6 mm) were punched into each plate. Each well of the compound preparation group was added 100 μL of the glycyrrhizic acid and *Isatidis folium* extract compound and 100 μL of *B. subtilis* cell-free supernatant. Each well in the antibiotic group was added 200 µL of penicillin/streptomycin solution (10,000 U/mL/10 mg/mL), and the cultures were incubated at 37 ℃ for 12 h. Each well of the control group was added 200 μL of LB liquid medium. The diameter of the inhibitory ring was measured with a millimeter scale, and the edge of the inhibitory ring was limited to the extent that no obvious bacterial growth occurred.

### Analysis of the effects of compound preparation on growth performance and immunity of Hu sheep

#### Animals experimental design.

*B. subtilis* was processed into a dry powder formulation by Daqing Shiheyuan Dairy Industry Co., Ltd., containing 1.0 × 10^11^ CFU/g viable bacteria. The composition of the compound preparation used in this study is shown in Table 1. The optimal proportion of each component in the formulation was determined in our preliminary study using orthogonal experimental design and range analysis. The compound preparation consisted of 40% glycyrrhizic acid, 20% *Isatidis folium* extract, and 40% *B. subtilis* powder.

**Table 1:** The composition of compound preparation.

Species	Kilograms
Glycyrrhizic acid	12
*Isatidis folium* extracts	6
*B. subtilis* powders	12
Total amount	30

Twenty-seven 45-days-old healthy male Hu lambs (body weight = 15.83 ± 0.41 kg) from Heilongjiang Tangfeng Animal Husbandry Co., Ltd., China, were randomly assigned to 3 dietary groups, each dietary group consisted of 9 animals, were housed in 3 pens with 3 sheeps in each. Randomization was performed in two stages. Briefly, Twenty-seven sheep were individually ear-tagged and numbered sequentially from 1 to 27. Two-stage randomization was applied. In the first stage, a random value between 0 and 1 was generated for each sheep using the “RAND(0,1)” function in Microsoft Excel. Animals were sorted by these random values and allocated into three experimental groups (9 sheep per group). In the second stage, within each experimental group, the 9 sheep were subjected to a second independent randomization using the same “RAND(0,1)” function. Sheep within each group were randomly assigned to 3 pens (3 sheep per pen). Group allocation was performed by a researcher independent of animal sampling and subsequent laboratory analyses. Three dietary groups including control (fed with the basal diet); compound preparation group 1 (CP1, fed basal diet supplemented with 25 g/kg compound preparation), and compound preparation group 2 (CP2, fed basal diet supplemented with 30 g/kg compound preparation). A 5 days adaption period was followed by 60 days of experimental period. The feed compositions and nutrient levels of experimental ration are shown in Table 2.

**Table 2:** Feed ingredients and nutrient levels of experimental diets (%).

Ingredient	Ratio
Maize	28.5
Distillers dried grains with solubles	9.0
Corn germ meal	5.5
Corn starch residue	3.0
Extruded soybean	13.0
Glutamic acid	3.0
Wheat bran	6.8
Soybean oil	0.3
Molasses	3.0
Sodium bicarbonate	0.5
Ammonium chloride	0.8
Mountain flour	1.3
Calcium hydrogen phosphate	0.2
Sodium chloride	1.0
Premix^1^	0.4
Peanut shell	8.7
Beanstalk	15.0
Total	100.0
**Items**	**Nutrient levels (DM basis)**
Metabolic energy /MJ/kg	11.4
Crude protein CP	18.2
Neutral detergent fiber NDF	32.9
Acid detergent fiber ADF	14.7
Calcium Ca	1.0
Phosphorus P	0.5

^1^The premix provides the following per kilogram of diet: iron 100 mg, manganese 135 mg, zinc 100 mg, cobalt 0.5 mg, copper 12.5 mg, selenium 0.3 mg, iodine 1.5 mg, vitamin A 14,000 IU, vitamin D 2,000 IU, and vitamin E 50 mg.

The test lambs were numbered and housed in semi-open sheds with good ventilation. The lambs were fed at 08:00 and 20:00 daily in 2 separate feedings. The health status and behavioral performance of the lambs were monitored daily. Compound preparation was mixed into the concentrate for feeding. Lambs were fed and watered ad libitum. Feed intake was recorded daily and lamb weights were weighed every 30 days for each lamb (27 animals total). Daily feed intake (DFI), daily weight gain (DWG) and feed to gain ratio (F:G) were calculated for each sheep. Disinfection, disinsection and immunization were conducted according to routine procedures of the farm.

No animals died before meeting criteria for euthanasia. On 60^th^ day of the experiment, two and a half hours after the morning feeding, three sheep per group were randomly selected and injected with Lumianning anesthetic (the main component: xylazine hydrochloride, 0.1 g/sheep, Huamu Animal Health Care Products Inc. Jilin. China) into their neck muscle. Sheeps achieved complete anesthesia at 0.5 h after administration (9 animals total), and blood samples of 5 mL were collected from each sheep via jugular vein puncture. The blood samples were incubated at 37 ℃ for 1 h; the supernatant was collected, centrifuged for 10 min at 3000 × g, and the serum was obtained. After blood collection in jugular vein, the lambs were euthanized by exsanguination under anesthesia. The colonic tissue, and colonic mucus samples were collected immediately. The intestinal mucus was obtained by scraping the mucosa of the colon with a slide. The mucus was placed in 2 mL Eppendorf tubes prefilled with 2 mL sterile PBS and incubated for 12 h at 4 ℃. After centrifugation at 3000 × g for 1 min, collection of supernatants was carried out, followed by their storage at −20 ℃ until use. The colon tissues were flushed with physiological saline, and 3 cm samples were taken and stored in RNA sample preservation solution (SevenShelter, Harbin, China) at −20 ℃.

#### Analysis of immunoglobulin content.

Serum IgG and immunoglobulin A (IgA), and colonic mucus SIgA were quantified by ELISA according to the manufacturer’s instructions (Jianglaibio, Shanghai, China).

#### Gene expression analysis.

Extraction of the total RNA was carried out from colon tissue using a commercial RNA extraction kit (Fastagen, Shanghai, China). cDNA was synthesized with oligo (dT) primers using a reverse transcription kit (TaKaRa, Dalian, China). Quantitative real-time PCR was performed on a qTower³G instrument (Analytik Jena, Germany) in 20 μL reactions following previously described amplification parameters [22]. Primer sequences are listed in Table 3 and were synthesized by Saisens Biotechnology Co., Ltd. (Harbin, China). β-Actin was employed as the internal control, and relative gene expression was calculated using the method of 2^⁻ΔΔCt^.

**Table 3:** Primer sequences of cytokines and intestinal barrier factors.

Gene	Primer sequence (5’-3’)	Accession number
β-actin	F- TGGACTTCGAGCAGGAGATGGC	JN033788.1
	R- CCGCTCATTGCCGATGGTGATG	
IL-1β	F- CTAAACCGGCTCAACCCAG	NM_001009465.2
	R- CGGGCAGGAGCAGCAT	
IL-4	F- CTGGAATTGAGCTTAGGCGTATC	Z11897.1
	R- GCGTACTTGTACTCGTCTTGGCT	
IL-6	F- GTGATGACTTCTGCTTTCCCTAC	NM_001009392.1
	R- TGCCAGTGTCTCCTTGCTGT	
IL10	F- CGAGATGCCAGCACCCT	NM_001009327.1
	R- GCTGTTCAGTTGGTCCTTCATT	
IL-17	F- TGTATCAATGCTGAAGGGAAGGT	AF166488.1
	R- CTTCTCCAGCCGGAAGGAGT	
IFN-γ	F- TTCAGAGCCAAATTGTCTCCTTC	NM_001009803.1
	R- CAGTTTCTCAGAGCTGCCGTT	
ZO-1	F- GCATGATGATCGCCTGTCCTACCT	XM_042235166.1
	R- CCCGCCTTCTGTATCTGTGTCTTCA	
Occludin	F- GGATCAGGGAATATCCACCTATCAC	AJ313191.1
	R-CTTTATTGACCTCATCAAGTTCTGCTTG	
β-catenin	F- TGCCCTGGTGAAGATGCTTGGTT	JX880035.1
	R- GCCAGCTAAACGCACTGCCATTT	
Muc2	F- TCCAGGGCTCCATCACGCTCAT	DQ152978.1
	R- GCAGCCGTTGTGCGAAATCTCC	

### Analysis of protective effects of compound preparation on lamb colitis

#### Study design.

The experiment lasted for 10 days. Eighteen 45-days-old healthy male Hu lambs (body weight = 16.24 ± 0.86 kg) from Heilongjiang Tangfeng Animal Husbandry Co., Ltd., China, were randomly assigned to three groups (n = 6 per group). Randomization was performed as follows: eighteen sheep were individually ear-tagged and numbered sequentially from 1 to 18. A random value between 0 and 1 was generated for each sheep using the “RAND(0,1)” function in Microsoft Excel. Animals were sorted by these random values and allocated into three experimental groups (6 sheep per group). Group allocation was performed by a researcher independent of animal sampling and subsequent laboratory analyses. In the control group, the sheep were fed a basal diet throughout the experiment; in the DSS group, sheep were fed the same basal diet for the first 7 days and were then given a single daily oral gavage of DSS at 4 g/kg body weight thereafter; in the compound preparation-DSS (CP-DSS) group, sheep were fed a basal diet supplemented with 25 g/kg compound preparation for the first 7 days, followed by the same daily DSS gavage regimen as the DSS group thereafter.

The test lambs were numbered and housed in semi-open sheds with good ventilation. The lambs were fed at 08:00 and 20:00 daily in 2 separate feedings. The health status and behavioral performance of the lambs were monitored daily. Diarrhea incidence was monitored daily.

No animals died before meeting criteria for euthanasia. On 3^th^ day of DSS administration, more than 80% of the sheep in the DSS group developed diarrhea, three animals per group were randomly selected and injected with Lumianning anesthetic (the main component: xylazine hydrochloride, 0.1 g/sheep, Huamu Animal Health Care Products Inc. Jilin. China) into their neck muscle. Sheeps achieved complete anesthesia at 0.5 h after administration (9 animals total), and then euthanized by exsanguination. Subsequently, the colonic tissues were dissected out and carefully rinsed with physiological saline. A 3 cm segment was preserved in RNA stabilization solution (SevenShelter, Harbin, China) at −20 °C for gene expression analysis, and a 5 cm segment was fixed in 4% paraformaldehyde for 48 h for histopathological analysis.

The remaining sheep in the CP-DSS and DSS groups were treated and rehabilitated by a veterinarian.

#### Gene expression analysis of cytokines and intestinal barrier factors.

Cytokines (IFN-γ, IL-1β, IL-4, IL-6, IL-10) and intestinal barrier factors (ZO-1, Occluding, Muc2, α-catenin) were quantified by quantitative real-time PCR, the experimental procedures were the same as those outlined above. Primer sequences are listed in Table 3.

#### Histopathological analysis.

Paraformaldehyde-fixed colon tissues were dehydrated, paraffin-embedded, sectioned, and stained with hematoxylin and eosin (H&E) following standard protocols [24]. Histopathological morphology was examined using an Olympus BX53M microscope. The histopathology evaluation criteria were as previously described [25].

### Statistical analysis

The data are expressed as the mean ± standard error of three replicates and were analyzed using GraphPad Prism version 5.0.1. Since the datasets conformed to Gaussian distribution and met homogeneity-of-variance assumptions, one-way ANOVA was adopted for comparison among three or more groups. Tukey’s HSD post-hoc test was further applied for all-pairwise multiple comparisons. *P < 0.05 was considered statistically significant, **P < 0.01 was considered highly significant.

## Results

### Glycyrrhizic acid and *Isatidis folium* extract do not affect the growth of *B. subtilis*

As shown in Fig 2, supplementation with 5%, 10%, or 15% concentrations of glycyrrhizic acid or *Isatidis folium* extract did not significantly affect the growth of *B. subtilis*.

**Fig 2:**
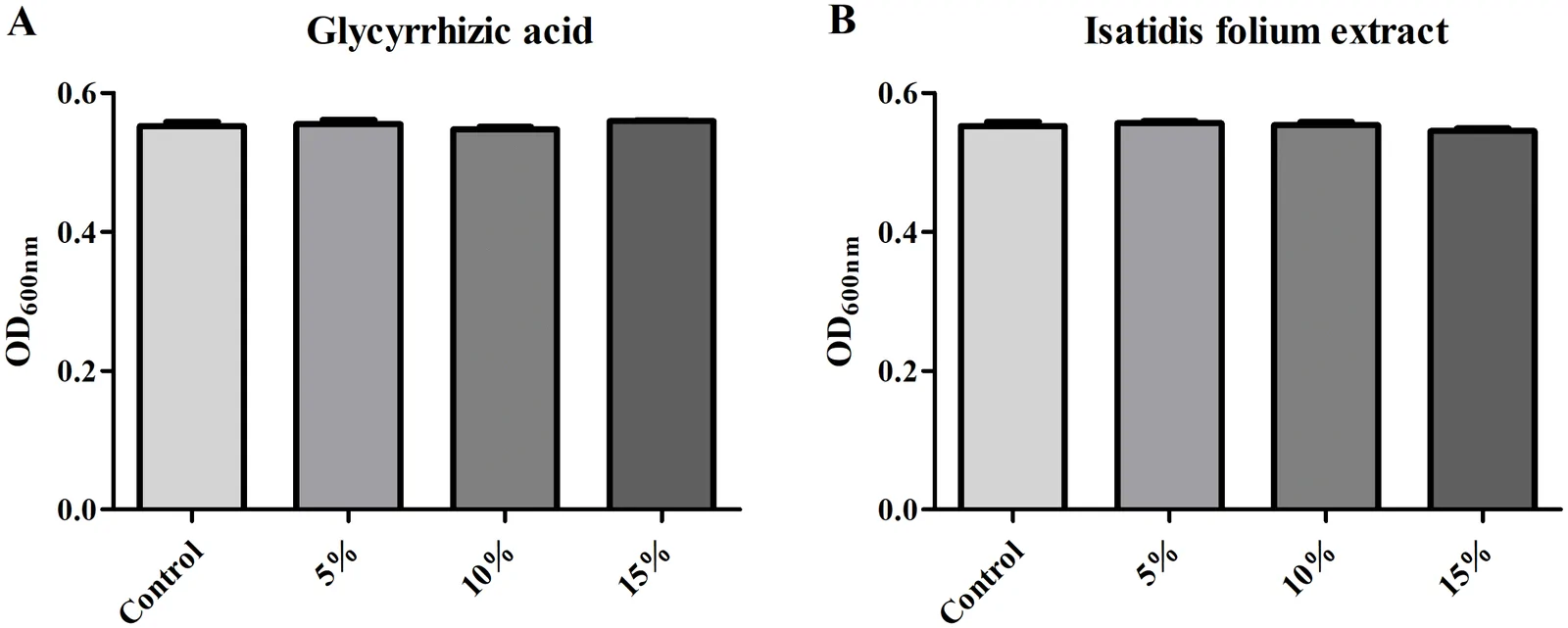
Effects of glycyrrhizic acid and *Isatidis folium* extract on the growth of *B. subtilis.* (A) Growth of *B. subtilis* in 5%, 10%, or 15% glycyrrhizic acid, respectively. (B) Growth of *B. subtilis* in 5%, 10%, or 15% *Isatidis folium* extract, respectively.

### The compound preparation inhibits the growth of pathogenic bacteria

As summarized in Fig 3, compared with control group, the CP exhibited significant inhibitory effects against *E. coli* (*P* < 0.01) and *S. aureus* (*P* < 0.01). However, the diameter of the inhibition zone was smaller than that of the antibiotic group. The CP showed no inhibitory activity against *S. typhimurium*.

**Fig 3:**
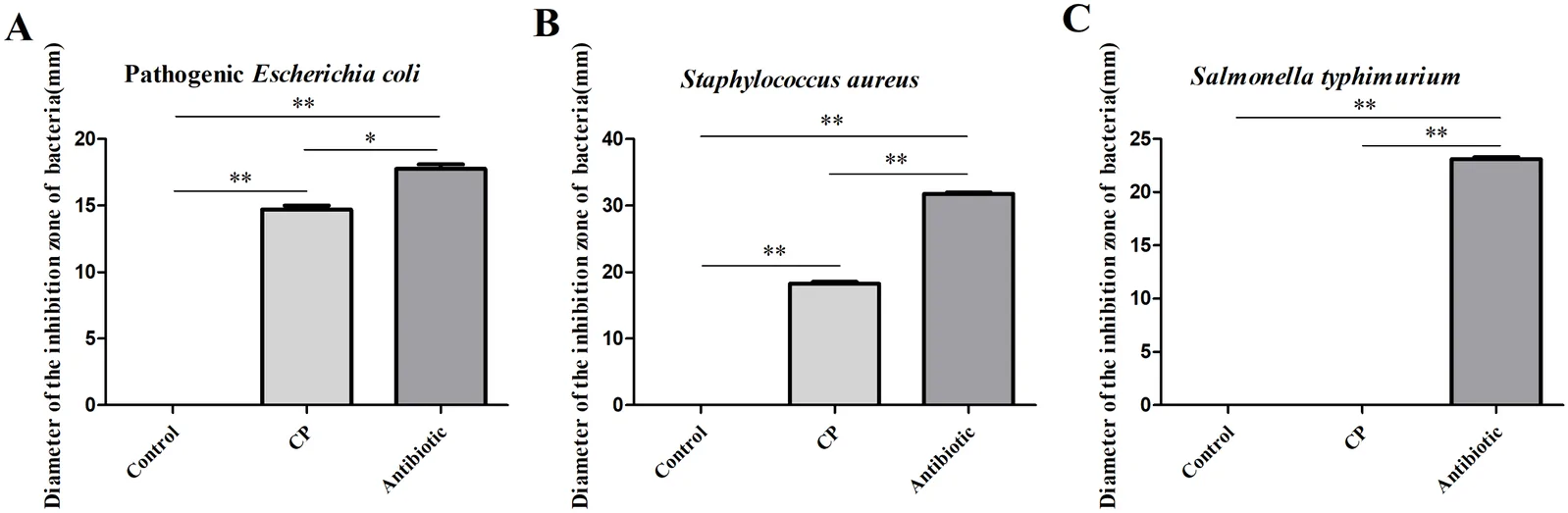
Effect of the compound preparation on the growth of pathogenic bacteria. The diameter of the inhibition zone of pathogenic *Escherichia coli* (A), *Staphylococcus aureus* (B), *Salmonella typhimurium* (C), * *P* < 0.05, ** *P* < 0.01.

### Performance

The treatments did not influence the results obtained for daily feed intake, feed to gain ratio (Table 4). However, the daily weight gain of sheep in the CP1 group was significantly increased, compared to the control group (*P* < 0.05), (Table 4).

**Table 4:** Effects of compound preparation supplementation on growth performance in Hu sheeps.

Item	Control	CP1	CP2
Initial BW, kg	15.72 ± 0.33	15.82 ± 0.45	15.69 ± 0.49
Day 60 BW, kg	29.00 ± 0.63	29.72 ± 0.87	29.10 ± 0.98
DFI, kg/day	1.62 ± 0.06	1.58 ± 0.09	1.59 ± 0.14
DWG, g/day	221.30 ± 8.57^a^	231.67 ± 10.07^b^	223.52 ± 11.41^ab^
F:G	7.31 ± 0.42	6.82 ± 0.27	7.12 ± 0.81

^a-b^Different letters in the superscripts of peer data indicate significant differences (*P* < 0.05), while no letters indicate no significant differences. BW = body weight; DFI = daily feed intake; DWG = daily weight gain; F:G = feed to gain ratio. n = 9 sheeps/group.

### The compound preparation enhances the immune function of Hu sheep

As shown in Fig 4, serum IgG levels and colonic mucosal SIgA levels in both CP1 and CP2 groups were significantly increased (*P* < 0.05) compared with the control group. No significant changes were observed in serum IgA levels (*P* > 0.05). Fig 5 shows that the relative mRNA expression of IFN-γ, IL-1β, IL-4, IL-6 and IL-10 was significantly higher in the CP1 and CP2 groups than in controls (*P* < 0.05). On the other hand, IL-17 expression levels did not differ significantly among groups (*P* > 0.05).

**Fig 4:**
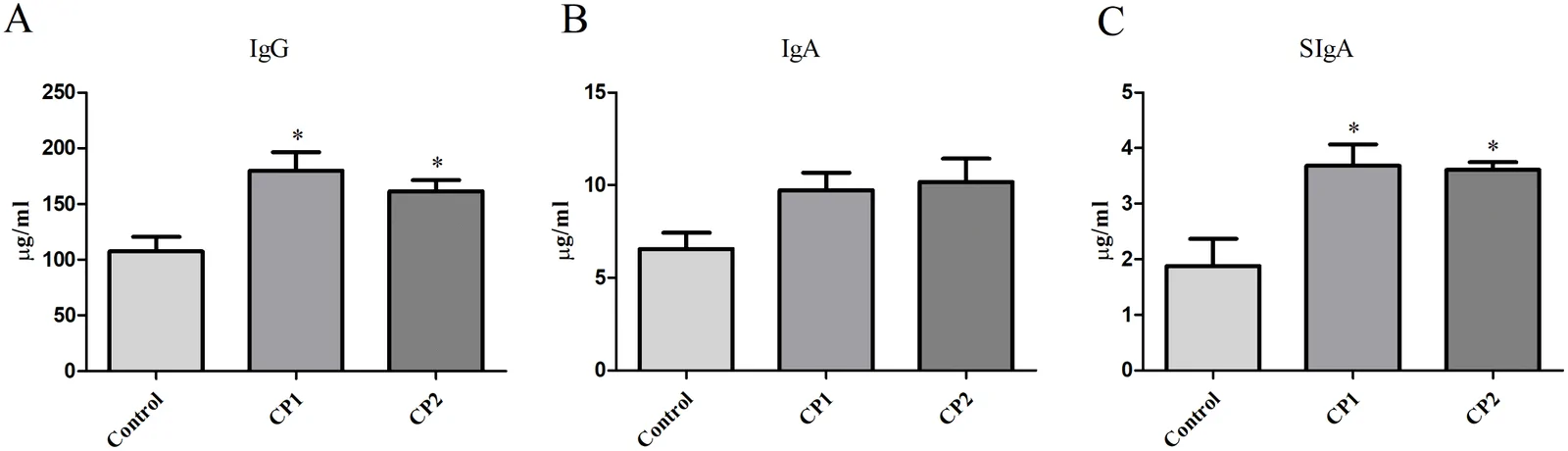
Effects of compound preparation on immunoglobulin contents of Hu sheep. The contents of IgG (A), IgA (B) in serum, and SIgA (C) in colon mucosa from each group, * indicate significant differences compared with the control group (*P* < 0.05).

**Fig 5:**
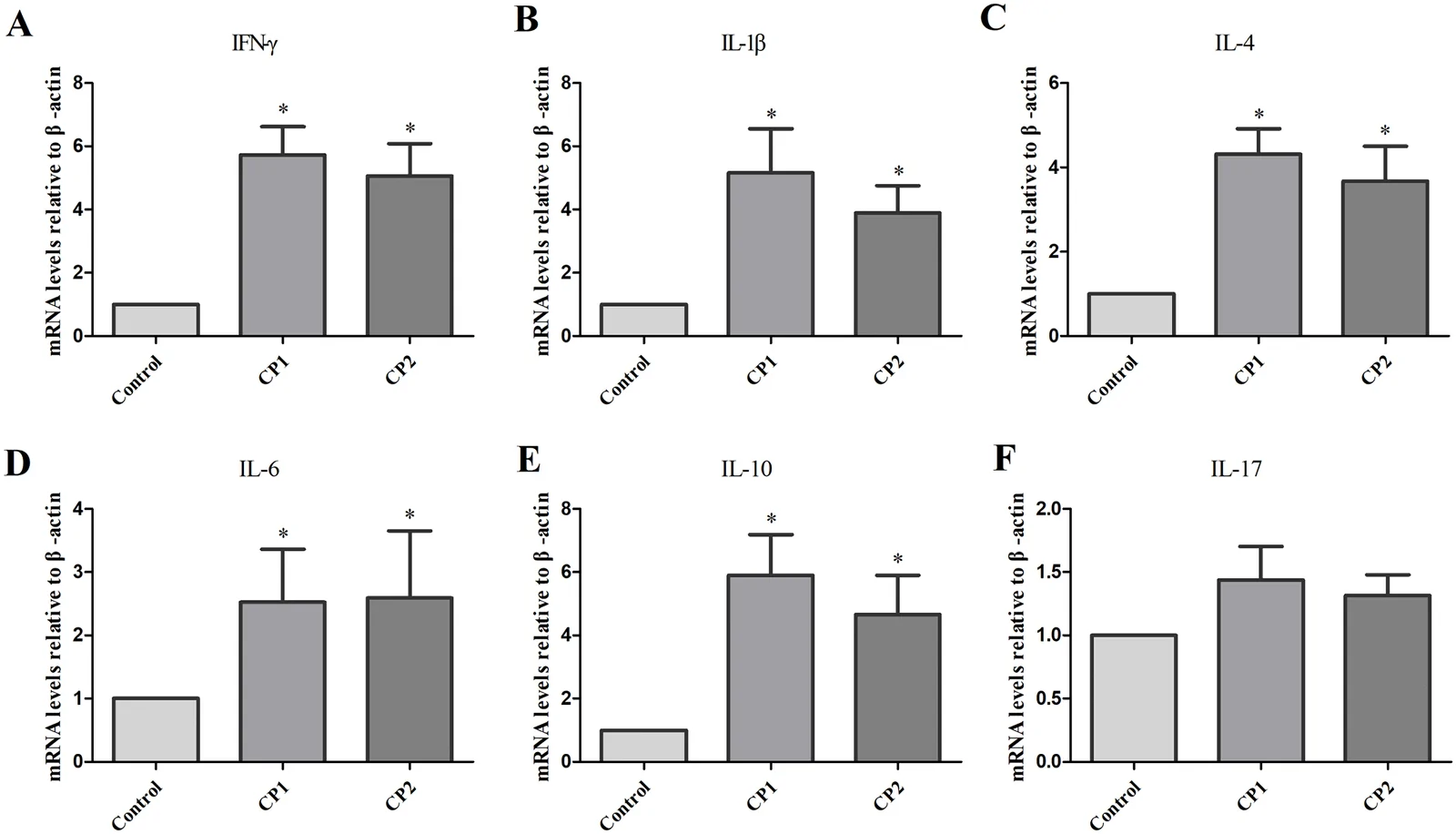
Effect of compound preparation on relative cytokine mRNA expression in colon tissue of Hu sheep. The relative mRNA expression of IFN-γ (A), IL-1β (B), IL-4 (C), IL-6 (D), IL-10 (E), IL-17 (F) in colon tissue from each group (n = 3 per group), * indicate significant differences compared with the control group (*P* < 0.05).

### The compound preparation ameliorates DSS-induced intestinal inflammation in Hu sheep

As shown in Fig 6, compared with the control group, the DSS group exhibited significantly elevated mRNA expression of proinflammatory cytokines IFN-γ, IL-1β, and IL-6 in colon tissue (*P* < 0.01). CP-DSS group significantly reduced the expression of these cytokines, while significantly increasing the expression of anti-inflammatory cytokines IL-4 and IL-10 relative to the DSS group (*P* < 0.01).

**Fig 6:**
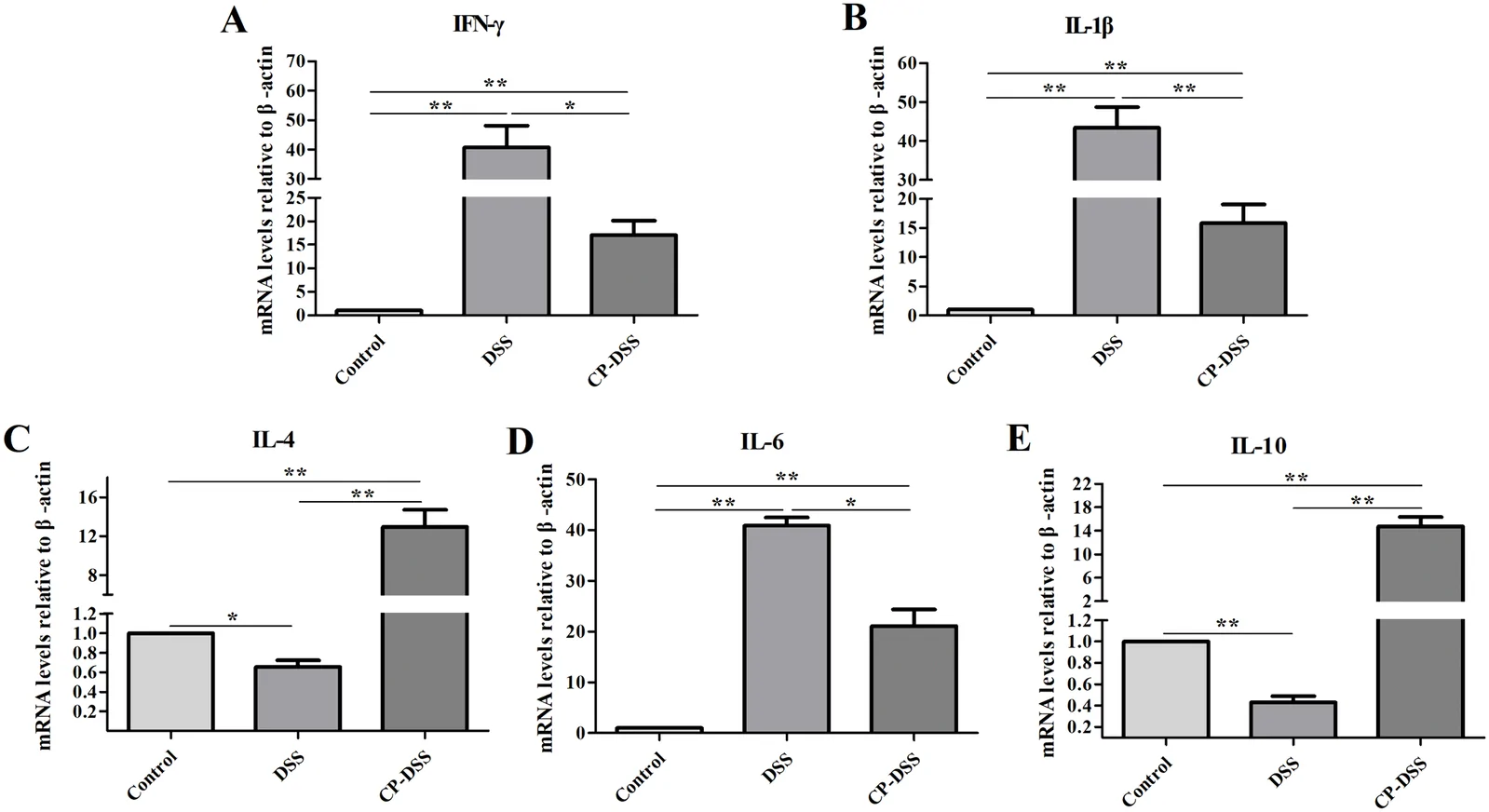
Effects of compound preparation on the mRNA expression of cytokines in the colon of Hu sheep. The relative mRNA expression of IFN-γ (A), IL-1β (B), IL-4 (C), IL-6 (D), IL-10 (E) in colon tissue from each group (n = 3 per group), * *P* < 0.05, ** *P* < 0.01.

### The compound preparation improves the colonic mucosal barrier in Hu sheep

As shown in Fig 7, the DSS group displayed significantly decreased mRNA expression of the mucosal barrier-related factors ZO-1, occludin, Muc2, and α-catenin compared with the control group (*P* < 0.01). However, expression of all four was significantly upregulated in the CP-DSS group relative to the DSS group (*P* < 0.05), indicating that the compound preparation effectively restored mucosal barrier integrity.

**Fig 7:**
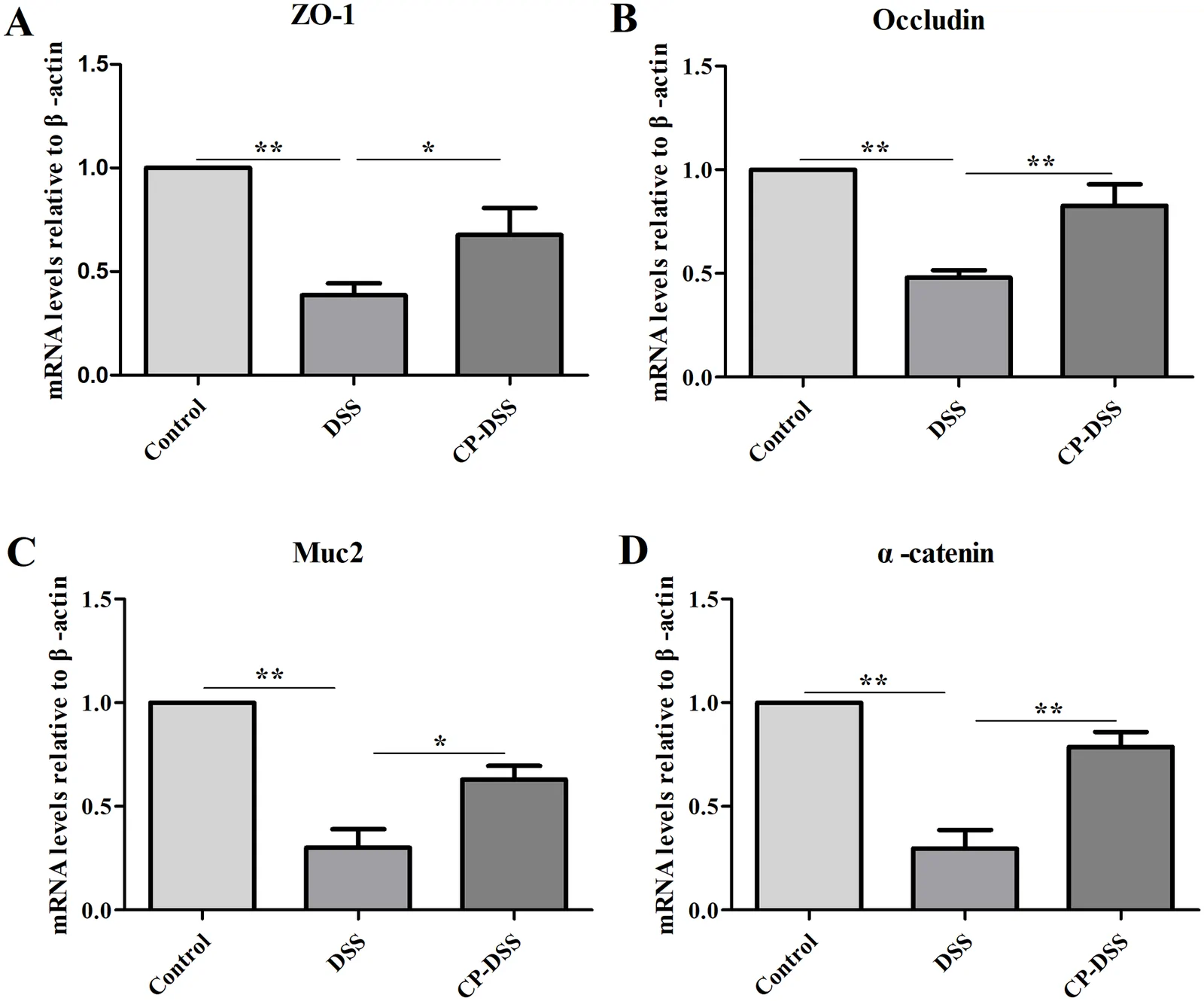
Effects of compound preparation on the mRNA expression of mucosal barriers in the colon of Hu sheep. The relative mRNA expression of ZO-1 (A), Occludin (B), Muc2 (C), α-catenin (D) in colon tissue from each group (n = 3 per group), * *P* < 0.05, ** *P* < 0.01.

### Compound preparation ameliorates DSS-induced colonic damage in Hu sheep

Histopathological examination revealed marked inflammatory injury in the colon of lambs in the DSS group. (Fig 8A). CP-DSS sheep exhibited less epithelium cell layer destruction, mucosa inflammation, and cell infiltration, which resulted in significantly lower histological scores than the DSS group (p < 0.05; Fig. 8B).

**Fig 8:**
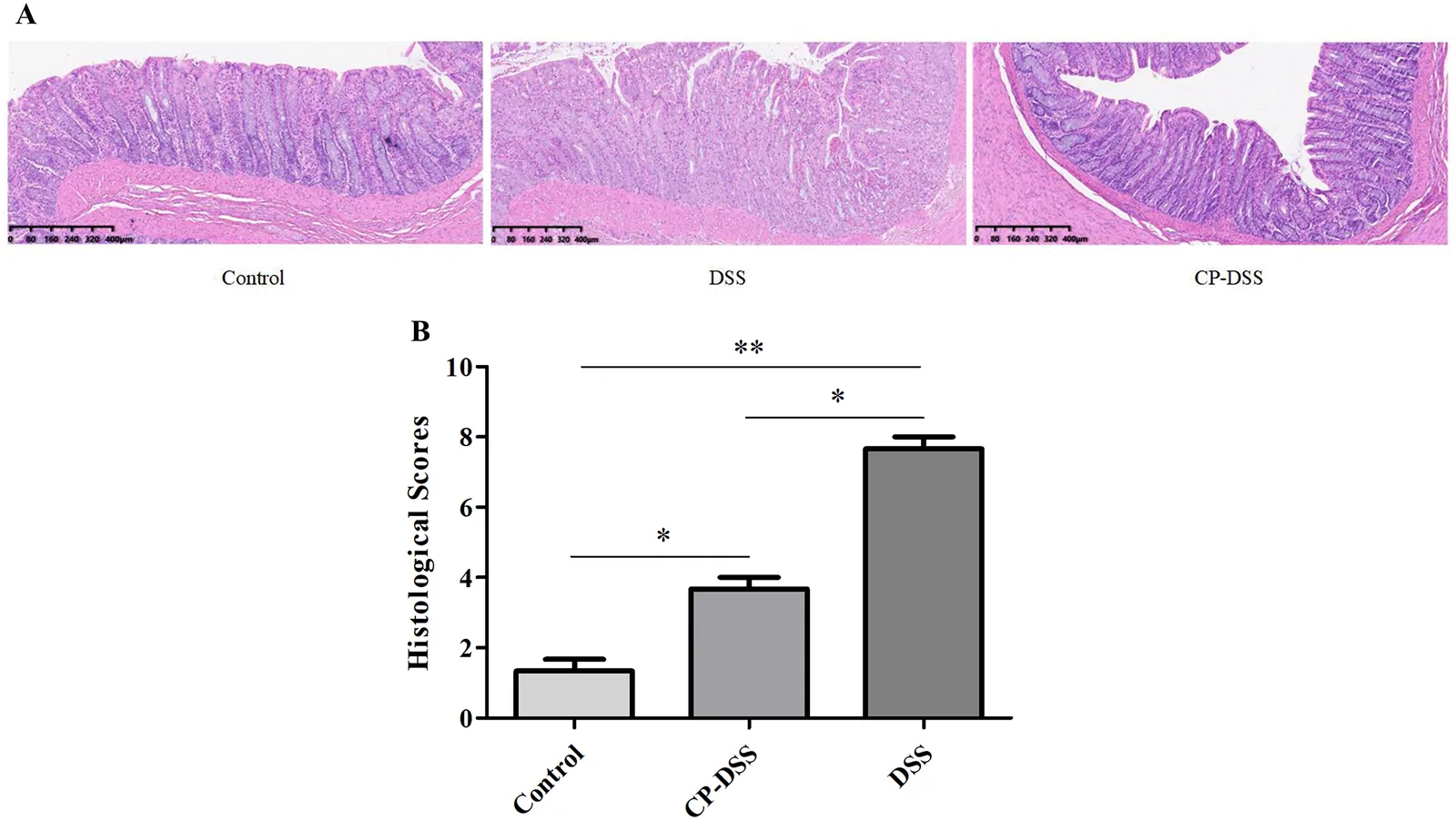
Compound preparation improves colon damage in DSS-treated Hu sheep. (A) Sections of colon tissues stained with hematoxylin and eosin. (B) Histopathology scores in the colonic tissues from each group (n = 3 per group), * *P* < 0.05, ** *P* < 0.01.

## Discussion

Abrupt environmental changes, separation from the mother, and dietary transitions often induce gastrointestinal disturbances such as diarrhea and enteritis in lambs [26]. Against the backdrop of the emergence of drug-resistant bacteria and the advocacy of antibiotic-free farming, TCM extracts and probiotics have gained significant attention as natural feed additives.

Glycyrrhizic acid, a principal active component of licorice (*Glycyrrhiza* spp.), exhibits anti-inflammatory, antiviral, and immunomodulatory properties and has been widely used as a food additive and therapeutic agent [27]. *Isatidis folium* (leaves of *Isatis indigotica*) also possesses antibacterial, anti-inflammatory, antiviral, antipyretic, and immunity-boosting activities [28]. Moreover, dietary supplementation with *B. subtilis* has been shown to enhance average daily gain and reduce inflammatory cytokine levels in meat sheep [29]. When used in compound with fermented Chinese herbal preparations, *B. subtilis* can also alleviate LPS–induced intestinal barrier disruption and enhance antioxidant enzyme activities [30].

Based on this evidence, glycyrrhizic acid, *Isatidis folium* extract, and *B. subtilis* were selected in this study to evaluate their combined effects on intestinal health and their potential protective efficacy against acute colitis in Hu sheep. Our preliminary results demonstrated that neither glycyrrhizic acid nor *Isatidis folium* extract inhibited the growth of *B. subtilis*, confirming the compatibility of these components within a single compound preparation. Furthermore, the preparation markedly inhibited the growth of *S. aureus* and *E. coli*, but not that of *S. typhimurium*. This differential effect may be attributed to the ability of glycyrrhizic acid to disrupt biofilm formation and alter the membrane permeability of *S. aureus* and *E. coli*, while exerting minimal impact on the cell membrane structure of *S. typhimurium* [31]. The disk diffusion assay is a semi-qualitative screening method. Its results are affected by sample solubility, the diffusion rate of active ingredients in agar, and molecular weight. This assay cannot generate quantitative MIC/MBC values and thus cannot precisely evaluate antibacterial activity, which constitutes a limitation of the present study. The broth microdilution method will be adopted in subsequent research for quantitative MIC-MBC determination. The inhibition zone diameters reported herein are only used as preliminary screening data for antibacterial activity. Since the primary purpose of this study was to optimize the compound formulation and explore its comprehensive efficacy in Hu sheep, we focused on investigating the overall biological activity of the compound preparation rather than designing a strict synergistic verification experiment with single-component control groups.

Dietary supplementation of 25 g/kg compound preparation, there were significant improvement in daily weight gain. The reason for this effect may be that *B. subtilis* can improve the ratio of serum fraction of albumin and albumin-globulin in sheep, and these globulins contribute to weight gain in sheep [32]. Another possible reason is that *B. subtilis* may remodel rumen microecology, promote fermentative energy production and the efficient conversion of nitrogen into microbial protein, and coordinately regulate lipid metabolism and redox homeostasis, thereby providing a metabolic basis for higher weight gain and improved feed efficiency in sheep [33]. The efficacy of feed additives is commonly assessed through clinical indicators such as immunoglobulin concentrations and cytokine profiles [34,35]. IgG plays a vital role in systemic immune defense against pathogen invasion, while IgA contributes primarily to mucosal humoral immunity. SIgA, present on mucosal surfaces of the gastrointestinal and respiratory tracts, neutralizes pathogens and prevents microbial adhesion and invasion through epithelial barriers [36,37]. It has been suggested that pathogenic microorganisms or toxic substances elicit pronounced host immune responses, often characterized by sharp elevations in proinflammatory cytokines, whereas probiotics or health-promoting additives induce moderate immune activation, shown by mild increases in cytokine and immunoglobulin levels [38]. In this study, dietary supplementation with the compound preparation significantly increased serum IgG levels and SIgA content in colonic tissue, accompanied by upregulated expression of several cytokines. These findings suggest that the preparation exerted an immunomodulatory effect in Hu sheep, enhancing both systemic and mucosal immunity.

Previous research demonstrated that *Isatidis folium* water extract inhibits the expression of TNF-α and IFN-γ, thereby ameliorating atopic dermatitis in mice [9]. Similarly, glycyrrhizic acid exhibits potent anti-inflammatory effects by downregulating the expression of high mobility group box 1 (HMGB1), Toll-like receptor 4 (TLR4), NF-κB, and TNF-α mRNA [39]. Glycyrrhizic acid also reduces the secretion of proinflammatory cytokines such as TNF-α and IL-1β, while increasing the anti-inflammatory cytokine IL-10, thus alleviating inflammation in septic mice [40]. Comparable immunoregulatory effects have been reported for *B. subtilis*, which can modulate cytokine profiles and support intestinal homeostasis [29]. In this study, a DSS–induced colitis model was established in lambs to evaluate the anti-inflammatory and intestinal protective effects of the compound preparation. Under inflammatory conditions, expression of IL-6, IL-1β, and IFN-γ increased significantly, and the affected animals exhibited characteristic pathological changes, including mucosal epithelial disruption, inflammatory cell infiltration, and mucosal hemorrhage. These observations were consistent with previous studies and confirmed the successful establishment of the DSS model [41]. Based on the analysis of the effects of CP on growth performance, immune function and colonic cytokine expression in sheep, the CP1 dosage (25 g/kg feed) was selected to investigate therapeutic efficacy. Compared with the DSS model group, the CP-DSS group displayed significantly reduced mRNA expression of IFN-γ, IL-1β, and IL-6 and increased expression of IL-4 and IL-10. The preparation elevates inflammatory cytokine expression under basal conditions yet suppresses it in DSS‑induced colitis. This moderate induction of inflammatory cytokines under basal conditions indicates that the preparation can activate immune responses and exert immunomodulatory functions. According to the findings reported by Yonghyun Lee et al. [42], an appropriate innate immune response is beneficial to the host, whereas excessive inflammatory responses induced by pathological injury and pathogenic invasion exert detrimental effects. In this study, the relative expression levels of inflammatory cytokines induced by the preparation under basal conditions were markedly lower than those in the DSS‑treated group. These results indicate that the preparation moderately modulates immune status. Under inflammatory conditions, however, the preparation could appropriately reduce the over‑expressed inflammatory cytokines in the colon and thereby exert partial anti‑inflammatory effects. The underlying mechanism may involve the preparation that targetedly modulates DSS‑overactivated macrophages and prevents their uptake by epithelial cells, consequently mediating anti‑inflammatory activity. It is well‑established that infiltration of lamina propria of intestinal mucosa by excessively activated macrophages and neutrophils is tightly associated with colitis [43]. Further investigations will be performed to elaborate the anti‑inflammatory mechanism of this preparation in future work. One limitation of this study is that we only detected mRNA levels of IFN-γ and IL-1β to evaluate intestinal inflammation. Further studies incorporating TNF-α, MPO and NF-κB protein activation will help better elucidate the mechanisms underlying intestinal inflammation in this model. Furthermore, the mRNA relative expression of intestinal barrier-related factors, including ZO-1, occludin, Muc2, and α-catenin, was markedly upregulated. These findings suggest that the compound preparation not only suppressed inflammation but also promoted intestinal mucosal repair, and that this repair was likely mediated by *B. subtilis*, which is known to enhance mucosal integrity and maintain intestinal epithelial barrier function by stabilizing tight junctions and modulating the gut microbiota [44]. The CP contains probiotic components, and modulation of gut microbiota represents one of its potential mechanisms of action. However, the present study mainly focused on indicators of intestinal inflammation and mucosal barrier, and gut microbiota sequencing analysis was not performed. Follow-up studies will further explore the regulatory effects of this compound preparation on the structure of gut microbiota and its metabolites. Histological examination further confirmed that the colonic mucosal inflammatory infiltration was significantly reduced and the injury alleviated in the CP-DSS group compared with the DSS group. Collectively, these results indicate that dietary supplementation with 25 g/kg of the compound preparation effectively mitigates DSS-induced colitis by inhibiting excessive inflammatory responses and preserving intestinal barrier integrity. Similar inclusion doses have been reported for combined Chinese herbal or probiotic preparations in previous animal disease‑model studies [45,46].

## Conclusion

In summary, the compound preparation composed of glycyrrhizic acid, *Isatidis folium* extract, and *B. subtilis* demonstrated multifaceted protective effects in Hu sheep. It inhibited the growth of pathogenic bacteria, enhanced immune and alleviated DSS-induced colonic inflammation and mucosal injury. Supplementation with 25 g/kg of the preparation effectively improved immune function. Overall, this preparation provides a promising strategy for the clinical prevention and treatment of lamb enteritis.
